# Neuronal Reprograming of Protein Homeostasis by Calcium-Dependent Regulation of the Heat Shock Response

**DOI:** 10.1371/journal.pgen.1003711

**Published:** 2013-08-29

**Authors:** M. Catarina Silva, Margarida D. Amaral, Richard I. Morimoto

**Affiliations:** 1 Department of Molecular Biosciences, Rice Institute for Biomedical Research, Northwestern University, Evanston, Illinois, United States of America; 2 Faculty of Sciences, Centre for Biodiversity, Functional and Integrative Genomics (BioFIG), University of Lisboa, Lisboa, Portugal; 3 Centre of Human Genetics, National Institute of Health, Lisboa, Portugal; Duke University Medical Center, United States of America

## Abstract

Protein quality control requires constant surveillance to prevent misfolding, aggregation, and loss of cellular function. There is increasing evidence in metazoans that communication between cells has an important role to ensure organismal health and to prevent stressed cells and tissues from compromising lifespan. Here, we show in *C. elegans* that a moderate increase in physiological cholinergic signaling at the neuromuscular junction (NMJ) induces the calcium (Ca^2+^)-dependent activation of HSF-1 in post-synaptic muscle cells, resulting in suppression of protein misfolding. This protective effect on muscle cell protein homeostasis was identified in an unbiased genome-wide screening for modifiers of protein aggregation, and is triggered by downregulation of *gei-11*, a Myb-family factor and proposed regulator of the L-type acetylcholine receptor (AChR). This, in-turn, activates the voltage-gated Ca^2+^ channel, EGL-19, and the sarcoplasmic reticulum ryanodine receptor in response to acetylcholine signaling. The release of calcium into the cytoplasm of muscle cells activates Ca^2+^-dependent kinases and induces HSF-1-dependent expression of cytoplasmic chaperones, which suppress misfolding of metastable proteins and stabilize the folding environment of muscle cells. This demonstrates that the heat shock response (HSR) can be activated in muscle cells by neuronal signaling across the NMJ to protect proteome health.

## Author Summary

The protein quality control machinery is responsible for preventing the accumulation of misfolded and damaged proteins and loss of cellular function. The capacity of cellular surveillance is limited however, leading to increased appearance of protein aggregates and risk for age-associated diseases. Here, we show that upregulation of acetylcholine receptors and moderate increased cholinergic activity leads to a calcium-dependent stress response that suppresses protein misfolding and restores homeostasis in *C. elegans* muscle cells. This involves *gei-11* knockdown-dependent upregulation of acetylcholine receptors, and the release of calcium into the cytoplasm of muscle cells through cell membrane and sarcoplasmic reticulum specific channels. Subsequently, activation of the heat shock factor 1 (HSF-1) leads to the expression of cytoplasmic chaperones that suppress misfolding of metastable and aggregating proteins, restoring folding and muscle function. This reveals a new non-canonical mechanism for the cell non-autonomous regulation of the heat shock response to ensure balance between cells in a metazoan.

## Introduction

Cellular health and organismal lifespan are critically dependent upon the fidelity of the proteome and the proteostasis network [1]. What are the molecular events that control proteostasis across tissues to activate protective responses at the cellular level to ensure organismal health? At the cellular level the heat shock response (HSR) and the unfolded protein response (UPR) respond to acute forms of proteotoxic stress with precise and rapid activation to restore homeostasis [2], [3]. In contrast to transient extreme stress, the chronic forms of protein damage and toxicity challenge the quality control machinery by their persistence and amplification effects on cumulative protein damage [4], [5]. How proteostasis monitors and responds to physiological stress is an area of active research [2], [6]–[8]. Yet, we know little about the regulation of stress responses under physiological conditions and at the organismal level. Elucidating these regulatory mechanisms is essential for a better understanding of diseases of altered protein conformation and age-related decline in cellular function [1], [9]–[11].

Much of our understanding of the HSR and the proteostasis network has come from studies using cultured cells and model organisms. The invertebrate animals *C. elegans* and *D. melanogaster* have been particularly amenable genetic models for identification of proteostasis components and modifiers of protein misfolding and toxicity [12]–[22]. These modifiers include cell autonomous factors such as molecular chaperones, proteasome subunits, components of the autophagy machinery, and the FOXO and heat shock factor 1 (HSF-1) transcriptomes that promote protein folding and clearance within the cell [20]. At the organismal level, the cell non-autonomous role of neuroendocrine signaling pathways and trans-cellular chaperone signaling has been shown to be important for lifespan, stress resistance, innate immunity and proteostasis [8], [9], . Moreover, tissue-specific regulation of mitochondrial function, including the electron transport chain and the mitochondrial UPR, was shown to affect the rate of aging [27]. Efforts to understand how cell autonomous and non-autonomous processes are integrated and co-regulated at the organismal level offer new genetic and pharmacological strategies to enhance the maintenance of proteostasis and health span.

In this study, we describe a new pathway for the heat shock response involving calcium signaling, in which *gei-11* knockdown-dependent upregulation of cholinergic receptor levels at the neuromuscular junction (NMJ) triggers activation of HSF-1. This reveals that under normal physiological conditions, the balance between cholinergic and GABAergic signaling at the NMJ regulates protein homeostasis in body-wall muscle (BWM) cells [28]. In contrast to the situation where complete inhibition of GABAergic signaling leads to overstimulation of muscle cells and dysfunction of post-synaptic cell proteostasis [29], we show here that a moderate increase in ACh receptors (AChR) at the NMJ, attained by down-regulation of *gei-11*, is beneficial to muscle cells. We demonstrate that there is a critical range of cholinergic activity at the NMJ leading to the Ca^2+^-dependent activation of HSF-1 and expression of molecular chaperones that results in an enhanced protective state in post-synaptic muscle cells.

## Results

### Identification of *gei-11*, a Genetic Modifier of Protein Aggregation and Toxicity

**Figure 1:**
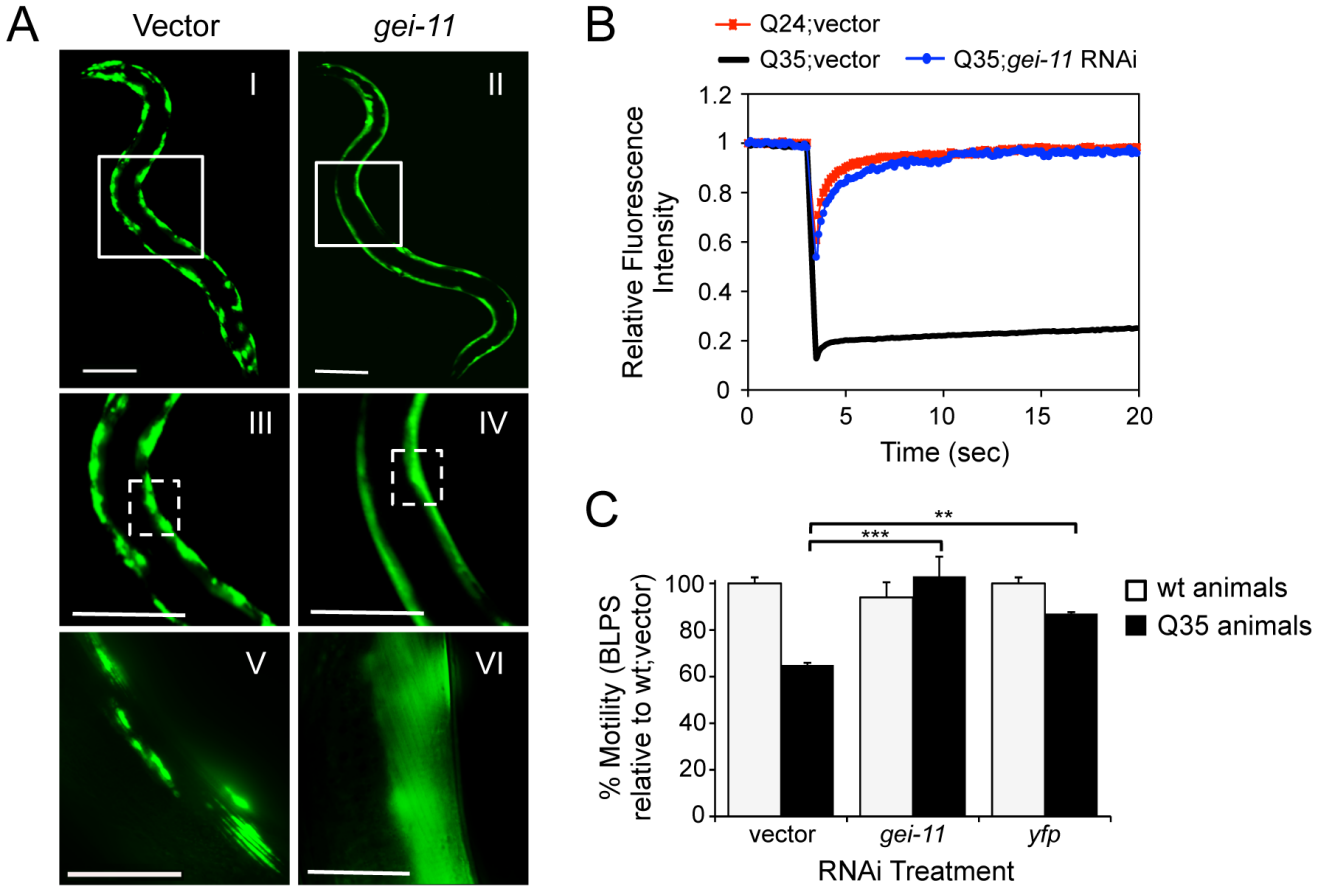
Knockdown of *gei-11* suppresses polyQ aggregation and toxicity. (A) *gei-11* RNAi suppressed Q35 aggregation in BWM cells of 6 day old animals, shown by the diffuse fluorescent pattern in II, IV and VI, in contrast to a foci-like pattern in the vector control I, III, V. Scale bar: 0.1 mm (I–IV), 0.025 mm (V–VI). Boxed areas correspond to the magnified images below. (B) FRAP analysis shows relative fluorescence intensity recovery at each time-point post-photobleaching. Control Q35 foci (in black; vector) revealed no fluorescence recovery, while *gei-11*-treated animals showed complete recovery of fluorescence (in blue), analogous to the soluble Q24 control (in red). Each curve represents an average of >12 independent measurements for *gei-11* RNAi, and >5 for the controls. (C) Motility assay for 6 day old Q35 and wt animals fed with vector, *gei-11* or *yfp* RNAi, measured in body-length-per-second and relative to wt speed in vector control (100%) (±SEM, Student t-test **p<0.01, ***p<0.001).

A genome-wide RNA interference (RNAi) screen performed in *C. elegans* for genetic modifiers of muscle proteostasis identified *gei-11* RNAi as a potent suppressor of polyglutamine (polyQ) aggregation in BWM cells (Figure 1A: II, IV, VI) [30]. Knockdown of *gei-11* also cross-protected against other aggregation-prone proteins, including polyQ37 that presents an earlier onset and more dramatic foci pattern (Figure S1A: I–IV) and mutant SOD1^G93A^ (Figure S1A: V–VIII). Suppression of Q35 aggregation by *gei-11* knockdown was achieved by maintaining the polyQ protein in a diffuse soluble state (Figure 1A–VI), as determined by fluorescence recovery after photobleaching (FRAP) (Figure 1B), and not by reducing the expression of Q35 mRNA or protein (Figure S1:B–D). Moreover, *gei-11*-mediated suppression of Q35 aggregation also led to the rescue of cellular toxicity as the motility of Q35 animals was restored to 100%, similar to knockdown of the polyQ transgene by *yfp*-RNAi (Figure 1C), without affecting the motility of wild-type (wt) animals. These results reveal that *gei-11* knockdown has potent suppressor activity of polyQ aggregation and toxicity.

### Enhanced Proteostasis Is Due to Increased Expression of L-Type AChR

The gene *gei-11* encodes the GEX-3-interacting protein 11 (GEI-11), a member of the Myb superfamily of transcription factors that is homologous to mammalian SNAPC4 (snRNA-activating protein complex subunit 4) [31]. *gei-11(tm6548)* is the only mutant allele available for this gene, and has a lethal phenotype (National Bioresource Project of Japan). *gei-11* has been proposed to have a negative regulatory effect on AChRs in *C. elegans* BWM cells, and is also expressed in head neurons, germ cells, somatic gonad, and intestine [32], [33].

**Figure 2:**
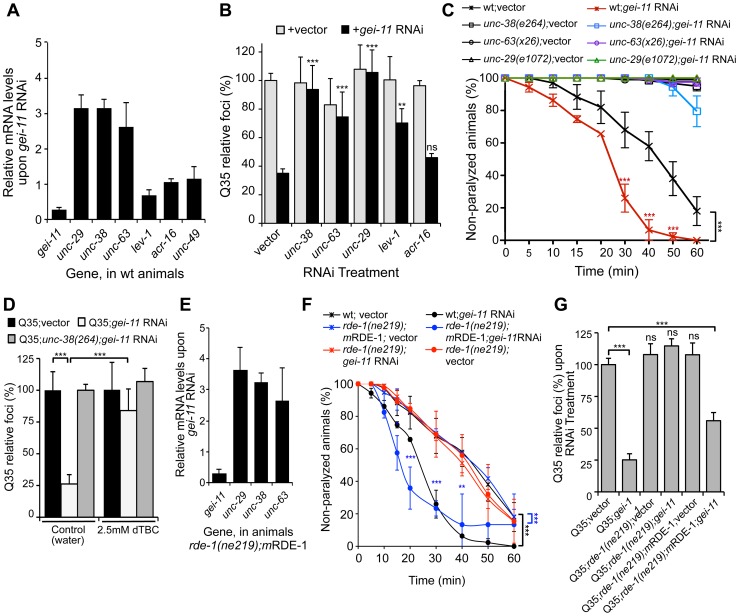
*gei-11* knockdown effect through regulation of cholinergic receptors at the NMJ. (A) Real-time qPCR analysis of AChR subunits *unc-29*, *unc-38*, *unc-63*, *lev-1* and *acr-16*, and GABA_R_ *unc-49*, in 6 day old wt animals fed with *gei-11* RNAi. Data are normalized to the levels of each gene on vector-treated wt animals (±SD). (B) Suppression of Q35 aggregation by *gei-11* RNAi was abolished by co-treatment with L-AChR (*unc-38, unc-63, unc-29*) but not with N-AChR (*acr-16*) subunits RNAi (±SD). Individual RNAi controls are shown in light grey (also see Table S1). (C) Cholinergic sensitivity assay: 5 day old animals treated with *gei-11* or vector RNAi were scored for paralysis on 1 mM levamisole plates (±SD). L-AChR mutant animals *unc-38(e264)*, *unc-63(x26)* and *unc-29(e1072)* were used as controls. Two-way ANOVA and Bonferroni test ***p<0.001 relative to vector control. (D) AChR antagonist dTBC (2.5 mM in water) prevented suppression of Q35 aggregation by *gei-11* RNAi (±SD). Q35;*unc-38(e264)* is a control for AChR-dependent effect. Student t-test ***p<0.001. (E) Real-time qPCR analysis of AChR subunits *unc-29*, *unc-38* and *unc-63* upon muscle-specific *gei-11* RNAi (*rde-1(ne219);m*RDE-1, 6 days old), relative to vector control (±SD). (F) Cholinergic sensitivity assay: 5 day old wt, *rde-1(ne219);m*RDE-1 and *rde-1(ne219)* animals treated with *gei-11* or vector RNAi were scored for paralysis on 1 mM levamisole plates (±SD). Two-way ANOVA and Bonferroni test ***p<0.001, **p<0.01, *p<0.05 relative to vector control. (G) Aggregation quantification upon *gei-11* RNAi in Q35, Q35;*rde-1(ne219);m*RDE-1 (muscle-specific RNAi) and Q35;*rde-1(ne219)* (impaired RNAi); shown as a relative % to Q35;vector (±SD). Student t-test ***p<0.001, ns/not significant.

In *C. elegans*, two types of ACh receptors, each with distinct subunit composition and pharmacology, are expressed at the NMJ [34]–[36]. To establish the specificity of *gei-11* downregulation on the expression of NMJ AChR subtypes, we monitored the expression of the L-type (levamisole-sensitive) AChR subunits *unc-29*, *unc-38*, *unc-63* and *lev-1*, and the N-type (nicotine-sensitive) homomeric AChR, *acr-16*. The effect of *gei-11* knockdown increased the expression of only the three essential subunits of the L-AChR (*unc-29*, *unc-38*, *unc-63*) by approximately 3-fold, and had no effect on the expression of N-AChR *acr-16* (Figure 2A). Likewise, *gei-11* RNAi did not affect the expression of the NMJ GABA receptor (GABA_R_ *unc-49*, Figure 2A). These results suggest that *gei-11* has a highly selective effect on the regulation of the L-type of cholinergic receptors.

To establish whether *gei-11* RNAi-mediated suppression of polyQ aggregation was dependent on elevated expression of the AChR, we performed double RNAi knockdown experiments and downregulated *gei-11* together with each of three L-AChR essential subunits (*unc-38*, *unc-63* or *unc-29*). The results showed that polyQ aggregation was unaffected when an essential L-AChR subunit was co-downregulated with *gei-11* (Figure 2B, Table S1). This was further confirmed using an L-AChR mutation (*unc-38(e264)*), that corroborated the results obtained with RNAi (Figure S2A). Moreover, *gei-11* co-downregulation with the non-essential L-AChR subunit *lev-1*, that only reduces receptor function [37]–[40], only weakened the *gei-11* effect on polyQ aggregation (Figure 2B). In contrast, double knockdown of *gei-11* with the N-AChR subunit *acr-16*, still suppressed Q35 aggregation (Figure 2B).

If the elevated expression of three essential L-AChR subunits results in increased L-AChR activity at the NMJ, this would predict increased sensitivity to levamisole, the cholinergic agonist that selectively activates L-AChR and causes hyper-contraction and paralysis [37]. Indeed, *gei-11* RNAi-treated wt animals showed a more rapid paralysis on levamisole plates relative to vector RNAi-treated animals (Figure 2C), consistent with the enhanced L-AChR activity at the NMJ. The specificity of levamisole sensitivity to L-AChR activity was confirmed using loss-of-function receptor mutations (*unc-38(e264), unc-63(x26), unc-29(e1072)*) [28], [35]. These animals were resistant to levamisole upon *gei-11* RNAi treatment (Figure 2C), supporting a *gei-11* effect dependent on AChR function. Similarly, mutant Q35;*unc-38(e264)* animals were resistant to levamisole compared to Q35 animals upon *gei-11* RNAi (Figure S2B). Consistent with specificity of *gei-11* to the L-AChR sub-type, RNAi-treated animals exposed to the agonist nicotine that targets N-AChR [34]–[36], did not show altered sensitivity compared to either wt or N-AChR mutant animals (*acr-16(ok789)*) (Figure S2C). Additional support for cholinergic-mediated effect on proteostasis was obtained using (+)-Tubocurarine chloride (dTBC), a potent inhibitor of AChR activity [28]. Consistent with our hypothesis, dTBC inhibited *gei-11* RNAi suppression of Q35 aggregation in a dose-dependent manner (Figure 2D, ); the specificity of this effect on L-AChR function was consistent with results obtained using the Q35;*unc-38(e264)* animals (Figure 2D).

The expression of GEI-11 is not restricted to muscle cells [33], therefore we determined whether the effect of *gei-11* RNAi and increased cholinergic receptor expression on muscle polyQ aggregation was a direct consequence of *gei-11* knockdown in muscle cells, rather than a downstream effect from another tissue. For example, *gei-11* RNAi did not have an effect on aggregation of polyQ expressed in the intestine (iQ44, Figure S1E). We next examined whether the enhancement of muscle proteostasis was a direct consequence of *gei-11* downregulation in muscle by using a *C. elegans* mutant strain in which the effects of RNAi are restricted to muscle cells: Q35;*rde-1(ne219);myo-3p-*RDE-1 (here referred to as *rde-1(ne219);m*RDE-1) [41], [42]. As a negative control, we employed a mutant strain impaired for RNAi in all cells, Q35;*rde-1(ne219)* (Figure S2E). Knockdown of *gei-11* in *rde-1(ne219);m*RDE-1 animals increased the expression of essential L-AChR subunits (>3-fold, Figure 2E), comparable to organism-wide *gei-11* RNAi (compare Figure 2A and C to Figure 2E and F). Likewise, aggregation in Q35;*rde-1(ne219);m*RDE-1 animals was suppressed by 50% upon *gei-11* RNAi relative to vector control, with no effects observed in Q35;*rde-1(ne219)* animals (Figure 2G). Taken together, these results show that suppression of protein aggregation in muscle cells is due to *gei-11* down-regulation in muscle cells and the consequent upregulation of AChR at the NMJ.

### Modulatory Effect of Cholinergic Receptors on Muscle Proteostasis

**Figure 3:**
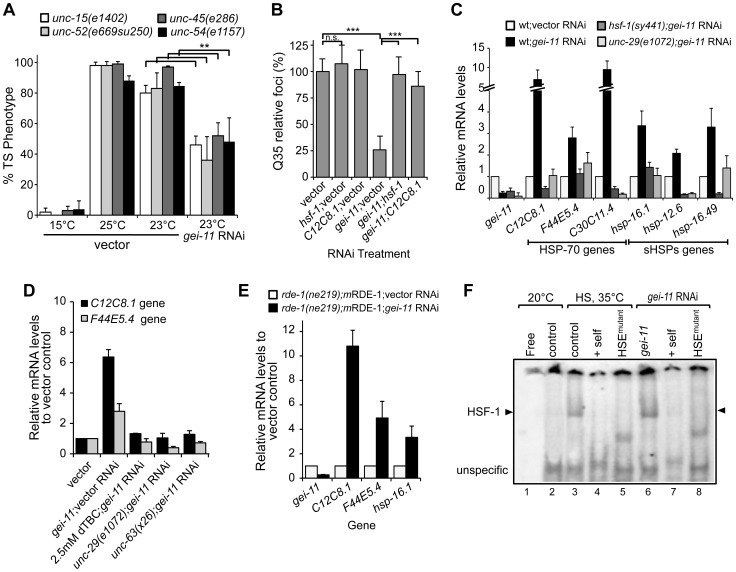
Rescue of proteostasis through HSF-1 activation. (A) *gei-11* RNAi (0.27±0.070 knockdown) suppressed the TS toxic phenotypes of UNC-15 (paramyosin; unc/slow movement), UNC-52 (perlecan, stiff paralysis), UNC-45 (myosin assembly, egg laying defect) and UNC-54 (myosin, paralysis). 15°C is the permissive temperature, 25°C is the restrictive temperature and 23°C is the temperature for RNAi (±SD, Student t-test **p<0.01). (B) *gei-11* double knockdown with *hsf-1* or *hsp-70* (*C12C8.1*) abolished the suppressor effect on Q35 aggregation (±SD, Student t-test ***p<0.001, see Table S1). (C) Real-time qPCR analysis of chaperone (Hsp-70 family *C12C8.1, F44E5.4, C30C11.4*; and small Hsps *hsp-16.1, hsp-12.6, hsp-16.49*) levels in wt, mutant *unc-29(e1072)* and *hsf-1(sy441)* animals, treated with *gei-11* RNAi (0.24±0.070 knockdown). Data are relative to wt;vector (±SD). (D) Real-time qPCR analysis of *hsp-70* (*C12C8.1, F44E5.4*) levels in 5 day old wt animals upon co-treatment with *gei-11* RNAi (0.16±0.082 knockdown) and AChR antagonist dTBC; and *gei-11* RNAi in the background of AChR mutants *unc-29(e1072)* or *unc-63(x26*), relative to wt in vector control (±SD). (E) Real-time qPCR shows upregulation of *hsp* levels upon muscle specific *gei-11* knockdown in *rde-1(ne219);myo-3p*-RDE-1 animals, relative to *rde-1(ne219);myo-3p*-RDE-1;vector (±SD). (F) Gel mobility shift analysis shows that *gei-11* RNAi induced HSF-1 DNA binding (lanes 6) in a similar way to heat shock at 35°C (lanes 3). Assay performed with a [^32^P]HSE oligonucleotide, HSE^mutant^ refers to a mutated oligonucleotide in the HSE (lanes 5,8), and *+self* refers to competition with 100-fold molar excess of unlabeled oligonucleotide (lanes 4,7). Control (lanes 2,3) refers to animals on vector RNAi.

To address whether the effect of *gei-11* knockdown-mediated suppression of polyQ aggregation was selective for this class of highly aggregation-prone species or reflected a more general enhancement of proteostasis in the BWM cells, we examined the effects of *gei-11* knockdown on the folding stability of four endogenous metastable proteins that function as folding sensors in muscle cells [4]. These metastable proteins exhibit temperature-sensitive (TS) phenotypes and harbor missense mutations in the paramyosin ortholog UNC-15, the basement-membrane protein perlecan UNC-52, the myosin-assembly assisting protein UNC-45, and myosin heavy chain UNC-54 [4], [11]. Thus, in animals held at the permissive temperature (15°C), all four TS-proteins are fully functional, whereas at the restrictive temperatures (23° and 25°C) these sensors misfold and each mutation results in an 80–100% loss of muscle function phenotype (Figure 3A control). However, when *gei-11* was downregulated at the restrictive temperature, the loss-of-function phenotype of each TS protein was decreased by 50–60% (Figure 3A). These results establish that downregulation of *gei-11* has general protective effects on the stability of multiple muscle cell proteins.

### Cholinergic-Mediated Activation of HSF-1 and Expression of Cytoplasmic Chaperones

To establish whether *gei-11* RNAi suppression of protein misfolding and aggregation in muscle cells is due to the expression of chaperones, we performed experiments in which *gei-11* was knocked-down together with either *hsf-1* or *hsp-70* (*C12C8.1*), to reveal that Q35 aggregation was no longer suppressed (Figure 3B). We monitored the expression of cytoplasmic chaperones of the HSP-70 family (*C12C8.1, F44E5.4* and *C30C11.4*) and small heat shock protein family (sHSPs *hsp-16.1*, *hsp-12.6* and *hsp-16.49*) in wt animals, and show that *gei-11* RNAi enhanced the expression of each chaperone gene from 2-to-10-fold (Figure 3C), a level that while substantial, is nevertheless much lower than observed when animals are exposed to acute heat shock treatment (>50-fold, Figure S3A). Moreover, the expression of these chaperones was not induced in the AChR mutant *unc-29(e1072*), or in HSF-1 mutant *hsf-1(sy441)* animals (Figure 3C). Therefore, upregulation of the proteostasis machinery by *gei-11* RNAi was absolutely dependent upon both cholinergic receptor activity and HSF-1. These results were further corroborated with the cholinergic antagonist dTBC and the L-AChR *unc-63(x26)* mutation (Figure 3D) that also prevented the upregulation of chaperones upon *gei-11* RNAi. The levels of *hsps* were also upregulated when *gei-11* was knocked-down specifically in muscle cells (*rde-1(ne219);m*RDE-1 animals, Figure 3E), providing additional support that the regulation of cholinergic receptors at the NMJ enhances muscle proteostasis.

To demonstrate directly that knockdown of *gei-11* induced the HSR, we monitored the activity of the heat shock transcription factor, HSF-1, using the electrophoresis mobility shift assay (EMSA) [43]. Knockdown of *gei-11* induced HSF-1 DNA-binding activity to the heat shock element (HSE) (Figure 3F: lane 6), similar to what was observed in animals exposed to heat shock (Figure 3F: lane 3). The specificity of HSF-1 binding was established using an *in vitro* competition reaction with excess unlabeled HSE (Figure 3F: lanes 4 and 7) and by using a mutant HSE radiolabeled oligonucleotide incapable of binding by HSF-1 (Figure 3F: lanes 5 and 8). These results demonstrate that *gei-11* knockdown activates HSF-1 transcriptional activity.

We further established the downstream regulatory effects of *gei-11* knockdown by examining the expression of other components of the proteostasis network, including the expression of the UPR-regulated ER chaperones (*hsp-3*, *hsp-4*, *dnj-7* and *ero-1*), metabolic stress FOXO/DAF-16 regulated genes (*sod-3* and *mtl-1*), and oxidative stress regulated genes (*hsp-6*, *gst-4* and *gcs-1*). As shown in Figure S3B, the expression of none of these other stress responsive genes was induced by *gei-11* knockdown in wt animals. Taken together, these results demonstrate that modulation of cholinergic receptors at the NMJ reprograms post-synaptic proteostasis through the activation of HSF-1 and the selective induction of cytoplasmic chaperones.

### Proteostasis Rescue by a Shift in the Balance of Cholinergic and GABAergic Signaling

**Figure 4:**
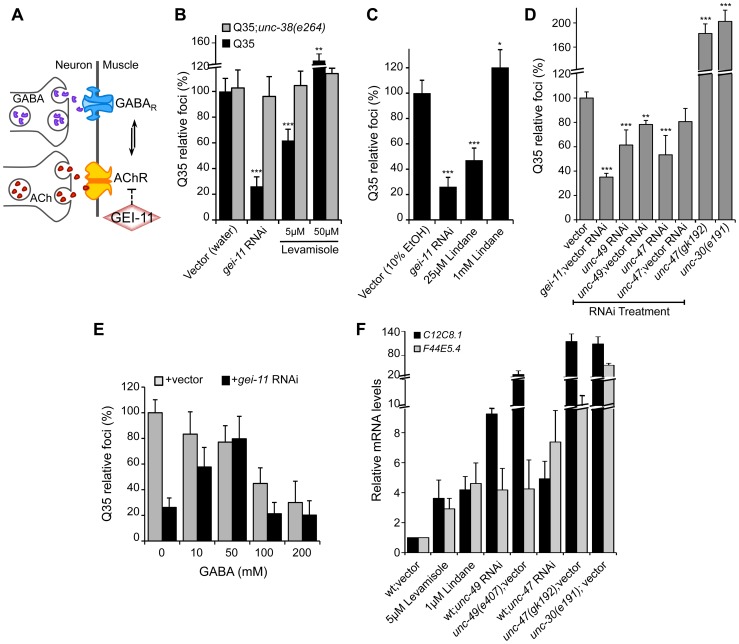
Modulation of AChR and GABA_R_ can restore post-synaptic folding. (A) At the *C. elegans* NMJ, the functional balance between GABA_R_ and AChR signaling regulates post-synaptic muscle function. (B) L-AChR activation with the agonist levamisole (in water) suppressed Q35 aggregation at 5 µM, but enhanced aggregation at 50 µM. Mutant AChR *unc-38(e264)* is a control for AChR-mediated effect. (C) Reduction in GABA_R_ function with lindane (in 10% EtOH) suppressed Q35 aggregation at 25 µM, and enhanced aggregation at 1 mM concentration (relative to EtOH control treatment). (D) Effect on Q35 aggregation by decrease in GABA with *unc-49* or *unc-47* RNAi, and by inhibition of GABA in *unc-47(gk192)* or *unc-30(e191)* mutant backgrounds. (E) Incubation with 50–200 mM GABA (in water) suppressed Q35 aggregation. GABA at 50 mM abolished the suppressor effect of *gei-11*, by “re-balancing” the GABAergic-cholinergic signaling. (F) Real-time qPCR analysis of *hsp-70* (*C12C8.1, F44E5.4*) levels in 5 day old wt animals upon treatment with ACh, levamisole or the GABA_R_ antagonist Lindane, or upon decrease in GABAergic signaling by either RNAi or mutant backgrounds of *unc-47(gk192)*, *unc-49(e407)* or *unc-30(e191)*. Student t-test **p<0.01 and ***p<0.001; data and statistics are relative to Q35;vector control (±SD) (RNAi controls: Table S1).

While upregulation of AChR at the NMJ induced the heat shock response and suppressed protein misfolding and toxicity in post-synaptic muscle cells, in previous studies we had observed that a null mutation in *unc-30*, the transcription factor that regulates the GABA operon, had the opposite result leading to enhanced aggregation in BWM cells [29]. This suggested that extreme cholinergic overstimulation was deleterious to muscle cell proteostasis [29]. Taken together with the results presented here for *gei-11*, we posit that there is a critical balance between the levels and activities of AChR and GABA_R_ (Figure 4A), with a physiological enhancement of AChR activity being proteo-protective and extreme overstimulation having proteotoxic consequences.

To address this hypothesis, we treated Q35 animals with the L-AChR agonist levamisole over a wide range of concentrations, and monitored aggregation in the post-synaptic muscle cells. At a low concentration of levamisole (5 µM) Q35 aggregation was suppressed by >40% (Figure 4B), whereas at higher levels (50 µM) that caused hyper-contraction, we observed the opposite result of 50–60% enhancement of aggregation (Figure 4B and S4). No effect on aggregation was observed in AChR mutant animals (Q35;*unc-38(e264)*) (Figure 4B). These results reveal that the beneficial effect on the folding environment is the consequence of a specific physiological range of cholinergic stimulation, and supports our conclusion that overstimulation has deleterious consequences on the folding environment of muscle cells.

We further explored how the imbalance between AChR and GABA_R_ activation at the NMJ affects muscle proteostasis by combining both genetic and small molecule agonists and antagonists probes. Reducing GABAergic activity by exposure to low concentrations (25 µM) of the GABA_R_ antagonist Lindane [29], [44]–[46] led to the suppression of Q35 aggregation (Figure 4C), comparable to a moderate increase of cholinergic signaling. Consistent with the genetic observations after cholinergic overstimulation [29], exposure to the highest concentrations of Lindane (1 mM), that cause BWM cells overstimulation, enhanced Q35 aggregation (Figure 4C). These results provide additional support that shifting the balance at the NMJ can enhance or harm proteostasis in the post-synaptic cell, in a magnitude of signal-dependent manner. To further test this hypothesis, we titrated GABA_R_ expression and GABA release at the synapse by using a combination of RNAi and specific loss-of-function mutations in the GABA pathway: *unc-30* (GABA synthesis), *unc-47* (GABA transport) and *unc-49* (GABA_R_). Knockdown of *unc-47* and *unc-49* suppressed Q35 aggregation, and this effect was less penetrant upon dilution of RNAi (1∶1) with vector RNAi (Figure 4D, Table S1). Conversely, eliminating GABA signaling in the *unc-30(e191)* and *unc-47(gk192)* mutants had the opposite effect (Figure 4D), consistent with previous results [29]. Finally, we altered the balance between cholinergic and GABAergic signaling by exposing *gei-11* RNAi-treated Q35 animals to increasing concentrations of GABA. At low concentrations (≤50 mM), GABA compensated for the *gei-11*-mediated increase in cholinergic signaling and prevented the suppression of Q35 aggregation, in a dose-dependent manner (Figure 4E). However, at very high GABA concentrations (50 mM–200 mM) this equilibrium shifted to the opposite direction and resulted in suppression of polyQ aggregation by GABAergic signaling (Figure 4E), also consistent with previous results [29]. The highest GABA concentrations tested (>200 mM, not shown) were very toxic and lethal to the animals. These results provide additional support to the importance of the magnitude of cholinergic and GABAergic signaling: an imbalance by either higher AChR or GABA_R_ transmits a signal that is interpreted by the muscle cell to activate a proteo-protective response; however, when this balance is severely disrupted, the consequence is proteotoxic.

Altering the balance between AChR and GABA_R_, also affected *hsp-70* expression. Exposure of wt animals to low levels of the AChR agonist levamisole (5 µM), or to the GABA_R_ antagonist Lindane (25 µM), resulted in elevated expression of the cytoplasmic *hsp70* family of chaperone genes (Figure 4F: *C12C8.1 and F44E5.4*), consistent with enhanced proteostasis. Likewise, and as expected, genetic reduction of GABA signaling using *unc-47* or *unc-49* RNAi (Table S1), which is equivalent to a moderate increase in cholinergic signaling, also upregulated a low level of *hsp-70* (<9-fold, Figure 4F) that restored the folding environment (Figure 4D). Consistent with our previous results, extreme overstimulation in the mutants *unc-47(gk192)*, *unc-49(e407)* or *unc-30(e191)* that no longer express GABA or GABA_R_, led to a massive upregulation of *hsp-70* (>50-fold, Figure 4F) that was not proteo-protective and resulted in elevated aggregation (Figure 4D), similar to the deleterious effects of acute heat shock treatment (Figure S3A).

Taken together, these results provide strong support for the importance of the regulation of the equilibrium between cholinergic and GABAergic signaling for optimal proteostasis. Within a critical physiological range, we show that increased AChR activity was beneficial and led to HSF-1-dependent moderate upregulation of cytosolic chaperones in muscle cells to establish a proteo-protective state. In contrast, extreme cholinergic overstimulation, whether obtained by genetics or small molecules, resulted in a dysfunctional proteostasis network that was deleterious.

### Upregulation of AChR Leads to Ca^2+^-Dependent Induction of the HSR

**Figure 5:**
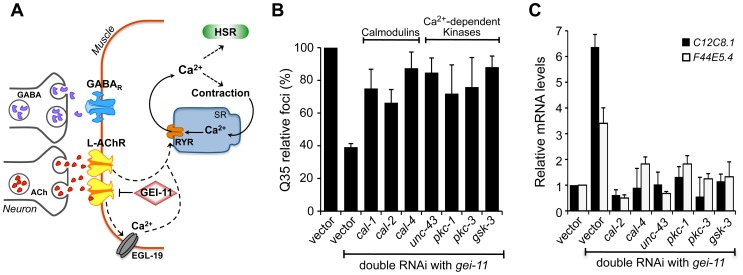
Ca^2+^-dependent kinases required for activation of the HSR and folding enhancement. (A) Cholinergic signaling at the NMJ activates muscle EGL-19 and Ca^2+^ flux into the cytoplasm of muscle cells, which further activates the ryanodine receptor (RYR) at the SR for muscle contraction. (B) Double knockdown of *gei-11* with calmodulin *cal-1*, *cal-2*, or *cal-4*; or Ca^2+^-dependent kinase *unc-43*, *pkc-1*, *pkc-3*, or *gsk-3*, prevented suppression of Q35 aggregation (±SD). % of foci are relative to Q35 in vector RNAi; Student t-test *p*<0.001. (C) Real-time qPCR analysis of *hsp-70* levels in wt animals upon double RNAi of *gei-11* with the indicated genes (±SD). Data are relative to vector-treated wt animals. *gei-11* levels were 0.23±0.101 upon RNAi, relative to vector sample.

The binding of ACh to receptors in BWM cells initiates a cascade of events that lead to the release of Ca^2+^ into the cytoplasm for muscle contraction [47] (Figure 5A). We therefore investigated whether the improvement in proteostasis through AChR-mediated HSF-1 activation was dependent on Ca^2+^ influx. Initially, we utilized the cell permeant Ca^2+^ chelator BAPTA [48], that alone (15 µM) had no effect on Q35 aggregation, but prevented *gei-11* RNAi induction of *hsp* chaperones and the subsequent suppression of polyQ aggregation (Figure S5A, B).

Activation of the HSR by increased levels of cytoplasmic Ca^2+^ also prompted us to examine the role of Ca^2+^-dependent kinases, as previous studies from our laboratory and others had identified serine residues of HSF-1 that are stress-inducibly phosphorylated by Ca^2+^-dependent kinases [49]–[55]. Therefore, we performed a candidate kinase screen to identify the kinases required for the *gei-11* RNAi suppression of Q35 aggregation (Figure 5B, Figure S5C) and induction of the HSR in wt animals (Figure 5C). This candidate screen identified calmodulins *cal-2* and *cal-4*; *unc-43/*CaMKII ortholog; *pkc-1*, *pkc-3*; and *gsk-3*; these genes correspond to the same mammalian kinases previously shown to regulate HSF-1. These results support that AChR upregulation initiates a cascade of Ca^2+^-signaling events leading to activation of HSF-1.

### EGL-19 and Ryanodine Receptors Regulation of Muscle Proteostasis

**Figure 6:**
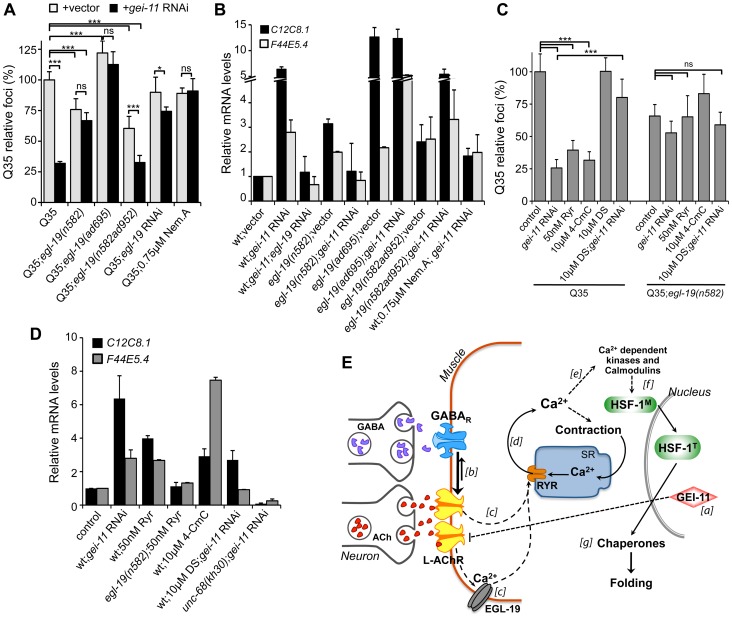
EGL-19- and RYR-mediated Ca^2+^ influx are components of the proteostasis rescue mechanism. Ca^2+^ relevance for *gei-11* effect on (A) Q35 aggregation (B) and *hsp-70* (*C12C8.1, F44E5.4*) upregulation, tested by employing a hypomorphic mutant *egl-19(n582)*, a weak hypermorph *egl-19(n582ad952)*, a hypermorph *egl-19(ad695), egl-19* RNAi (control RNAi in Table S1) or the specific EGL-19 antagonist Nemadipine A (0.75 µM, DMSO). Student t-test *p<0.05, ***p<0.001, ns/not significant; data relative to vector control or control in DMSO (±SD). (C) The RYR agonists ryanodine (50 nM, EtOH) and 4-CmC (10 µM, water) suppressed Q35 aggregation in a similar way to *gei-11* RNAi, but were less efficient in Q35*;egl-19(n582)* hypomorphic mutant animals. Treatment with the RYR antagonist DS (DMSO) together with *gei-11* RNAi prevented suppression of Q35 aggregation. Student t-test ***p<0.001, ns/not significant; data relative to vector control in respective compound % solvent (±SD). (D) Real-time qPCR analysis of *hsp-70* (*C12C8.1, F44E5.4*) levels: RYR agonists Ryr (50 nM) and 4-CmC (10 µM) up-regulated *hsp-70* in wt animals but not in mutant *egl-19(n582*) animals. Chaperone induction by *gei-11* RNAi was prevented in the RYR mutant (*unc-68(kh30)*) and by co-treatment with DS (±SD). *gei-11* levels were 0.27±0.150 upon RNAi, relative to vector sample. (E) Model for *gei-11* modulation of proteostasis in BWM. [a] Knockdown of *gei-11* by RNAi leads to an increase in L-AChR expression at the NMJ (dashed line: proposed genetic interaction). [b] This causes a shift in the cholinergic/GABAergic signaling at the NMJ towards higher (thick arrow) excitatory signaling into the muscle. ACh binding to AChRs activates the VGCC EGL-19. [c] Depolarization, conformational changes and Ca^2+^ influx through EGL-19 triggers the opening of RYR at the SR and further release of Ca^2+^ into the cytosol [d]. Ca^2+^ activates signaling cascades to promote muscle contraction [e], HSF-1 activation [f ] and expression of cytosolic chaperones that rescue protein folding in the cytosol [g]. Dashed lines represent proposed and simplified sequence of events.

Cholinergic activity at the NMJ leads to activation of the muscle voltage-gated Ca^2+^ channel (VGCC), EGL-19, and flux of Ca^2+^ into the cytoplasm [47] (Figure 5A). We therefore determined the role of EGL-19 activity on muscle proteostasis and polyQ aggregation using a partial (30% reduction) loss-of-function mutant (*egl-19(n582)*), a weak hypermorphic mutant (*egl-19(n582ad952)*), and a stronger hypermorphic mutant (*egl-19(ad695)*) in the background of Q35 [47], [56]. Our results showed that the magnitude of EGL-19 activity, that regulates Ca^2+^ flux into the muscle, had opposing effects: the strongest hypermorphic mutant *ad695* enhanced Q35 aggregation, whereas the weak hypermorphic *n582ad952* and weak hypomorphic *n582* mutants both suppressed Q35 aggregation (Figure 6A) [29]. Consistent with these effects on aggregation, moderate levels of chaperone upregulation (3-fold) were detected in animals where Ca^2+^ suppressed aggregation (*egl-19(n582)* and *egl-19(n582ad952)*; Figure 6B). By comparison, much higher levels of *hsp-70* (*C12C8.1, F44E5.4*; 15-fold) were observed in animals with Ca^2+^-mediated enhanced aggregation (*egl-19(ad695)*, Figure 6B). These results reveal a consistency between modulation of cholinergic signaling and Ca^2+^ influx on muscle cell folding environment, reflected by the effect on aggregation. Whereas a mild imbalance in Ca^2+^ influx, achieved with the weak hypermorph and hypomorph mutants, activated a protective HSR (corresponding to a moderate upregulation of *hsp-70*), the EGL-19 strong hypermorphic mutation, resulted in a much larger imbalance in signaling and accentuated stress response (corresponding to higher levels of *hsp-70*). These results provide further support for the importance of a critical physiological stimulation range to establish proteostasis.

To determine whether the enhanced folding capacity regulated by *gei-11* knockdown was dependent on the VGCC, we treated Q35;*egl-19* mutant animals with *gei-11* RNAi (Figure 6A). Knockdown of *gei-11* in hypomorphic *egl-19(n582)* mutant animals had no effect on Q35 aggregation (Figure 6A) or *hsp-70* expression (Figure 6B). This revealed that Ca^2+^ flux through EGL-19 was required for the beneficial effects of *gei-11* knockdown on protein homeostasis. These results were further supported by chemical-genetic approaches using *egl-19* RNAi and the EGL-19 inhibitor Nemadipine A [57] that block the *gei-11* RNAi effect on muscle proteostasis (Figure 6A, B, S6A, Table S1). Consistent with these observations, the effect of the weak hypermorphic mutant *egl-19(n582ad952)* was additive to the beneficial effects of *gei-11* RNAi on folding (Figure 6A, B), whereas the stronger hypermorphic mutant *egl-19(ad695)* effect on Ca^2+^ levels was deleterious (Figure 6A, B). These results establish the role of EGL-19 and Ca^2+^ influx function downstream of AChR upregulation to the rescue of post-synaptic proteostasis.

Activation of the VGCC, and the flux of Ca^2+^ into the cytoplasm of muscle cells, triggers the opening of the ryanodine receptor (RYR) at the sarcoplasmic reticulum (SR), releasing additional Ca^2+^ into the cytosol (Figure 5A) [58], [59]. We examined the contribution of RYR to induction of the HSR by stimulating RYR activity to mimic the effect of enhanced cholinergic signaling at the NMJ. Ryanodine (Ryr), a plant alkaloid with high affinity to the RYR, is a pharmacological agent widely used to study intracellular Ca^2+^ signaling in muscle cells [58], [59]. At low (nM) concentrations, Ryr acts as an agonist and sensitizes RYR channels to activation by Ca^2+^ [60], [61]. Treatment of Q35 animals with Ryr (50 nM) caused suppression of aggregation (Figure 6C) and upregulation of *hsp-70* (Figure 6D), and this effect was reduced in the background of the hypomorphic mutant *egl-19(n582)* (Figure 6C), supporting the model where enhancing both Ca^2+^ channels has beneficial effects on proteostasis. As observed for the natural agonist ryanodine, the clinically-used small molecule RYR activator 4-Chloro-m-cresol (4-CmC) [62] also up-regulated *hsp-70* levels and significantly reduced Q35 aggregation by more than 60% (Figure 6C, D, S6B). At high concentrations of 4-CmC (>1 mM), we only observed toxicity and no effect on aggregation (Figure S6B). These results establish that Ca^2+^ release by the RYR is involved in the enhancement of folding in muscle cells.

Recognizing that Ca^2+^ regulates many signaling cascades, we examined the specificity of *gei-11* RNAi-dependent Ca^2+^ release by the RYR on induction of *hsp-70* and suppression of polyQ aggregation, by testing a RYR-specific antagonist and a RYR mutant. We employed dantrolene sodium (DS), a clinically relevant muscle relaxant that selectively targets RYR and blocks Ca^2+^ release from the SR during muscle contraction [62], [63]. This antagonist prevented induction of *hsp-70* and suppression of Q35 aggregation by *gei-11* RNAi (Figure 6C, D, S6C). Similar results were obtained with the RYR mutant *unc-68(kh30)* (Figure 6D) [58]. From these observations, we conclude that Ca^2+^ flux from the RYR-SR is an important component of this new signaling pathway regulating muscle HSR. Finally, treatment of *egl-19* hypomorphic mutant animals (Q35;*egl-19(n582)*) with the RYR modulators had no significant effect on aggregation (Figure 6C) supporting the epistatic relationship of the two Ca^2+^ channels (Figure 6E). Taken together, these results demonstrate that the downstream events of AChR upregulation involve Ca^2+^-dependent activation of the HSR, and establish a new proteo-protective state in BWM cells with enhanced folding capacity (Figure 6E).

## Discussion

We demonstrate that cholinergic-dependent calcium signaling across the synaptic junction induces an atypical heat shock response that promotes protein homeostasis and suppresses misfolding and aggregation in post-synaptic muscle cells. These molecular events are dependent upon HSF1, but are distinct from the classical HSR regulated by transient exposures to acute heat shock stress. The key feature of this novel neuromuscular stress signaling mechanism, centers around the balance between cholinergic and GABAergic signaling at the NMJ. The importance of balanced signaling is highlighted by observations that muscle overexcitation caused by the complete absence of GABA is deleterious to the folding environment and results in a proteotoxic cellular environment [29]. Our present studies provide additional support for the importance of neuronal signaling in the control of somatic cell protein homeostasis, demonstrating that signaling balance at the NMJ can be perturbed to have either beneficial or detrimental consequences on the HSR-dependent proteome stability.

The identification of *gei-11* as a new genetic modifier of protein folding reveals a new strategy by which metazoans ensure proteostasis across tissues. Our biological observations suggest that the regulation of receptor expression in muscle cells can initiate a protective mechanism against stress and degeneration, such as age-dependent sarcopenia [11], [64], [65]. These results also suggest that neuronal signaling control of post-synaptic receptor function can achieve the same outcome. This may be highly relevant for complex pathologies, including neurodegenerative diseases and other neuromuscular disorders, where scenarios of protein misfolding initiated at one tissue have both autonomous (cell or tissue specific) and non-autonomous (inter-cellular) consequences on cellular function and organismal health. For example, neurodegeneration leads to muscle weakness and paralysis in motor neuron disorders such as ALS, hereditary spastic paraplegia, and spinal muscular atrophy [66]. Consequently, an understanding of the regulatory signaling cascades that trigger protective responses across tissues is of fundamental importance to delay or prevent the organismal collapse of proteostasis [22]. Modulation of signaling events at the NMJ to rescue muscle function, as described here upon knockdown of the gene *gei-11*, could suggest novel therapeutic targets for proteostasis maintenance with possible benefit for the patients suffering from somatic wasting diseases. Overall, it emphasizes the importance of dissecting neuronal signaling pathways that affect organismal stress responses and cellular function.

We propose that induction of the HSR by physiological regulation of cholinergic receptors reveals a new class of regulatory pathways of HSF-1 and chaperone networks that is distinct from the classical activation of the HSR. *gei-11* was identified from a genetic screen for proteostasis regulators that enhanced the cellular folding environment [30], and found to modulate AChR levels. The levels of ACh and GABA that activate the HSR are in contrast with the extreme imbalance and overstimulation of muscle cells caused by the absence of GABA, leading to proteotoxicity in the muscle cells [29]. An intermediate increase in cholinergic signaling at the NMJ, whether by genetic or small molecule upregulation of AChR activity or downregulation of GABAergic signaling, led to selective activation of the HSR and rescued folding capacity in muscle cells. As for cholinergic signaling, the folding rescue effect of Ca^2+^ influx in muscle cells homeostasis also occurs at a critical range. The activation of both EGL-19 and RYR channels by cholinergic signaling (Figure 6E) led to increased levels of cytoplasmic Ca^2+^ and activation of HSF-1 and chaperones that were physiologically beneficial, and well below the deregulated levels of signaling of these pathways that cause proteotoxicity. These results shift the emphasis from extreme environmental forms of stress to a new view on the roles of physiologically relevant *in vivo* stress signaling pathways regulation.

Aging and chronic stress challenge the cellular quality control systems by the accumulation of misfolded toxic proteins. Our findings strongly suggest that control of HSR and proteostasis, at the cellular level and at cell-non-autonomous level through neuronal signaling, are critical mechanisms in the cellular challenge to activate proteo-protective pathways and maintain homeostasis at the level of the organism. “Fine-tuning” of post-synaptic receptor expression, and therefore regulation of neuronal cholinergic signaling within physiologically relevant levels, may provide a potential strategy to enhance the functional properties of the proteostasis network. Our results contribute to the growing understanding of the properties of stress response networks, as an integrated organismal response to diverse challenges to the health and lifespan of the organism.

## Materials and Methods

### *C. elegans* Strains

Animals were maintained according to standard methods, at 20°C on nematode growth media (NGM) with OP50 *E. coli* [67]. The strains utilized in this work were previously described: wild-type (wt) Bristol strain N2; polyQ strains Q24 AM138 (rmIs130[P*_unc-54_*::*q24*::*yfp*]II), Q35 AM140 (rmIs132[P*_unc-54_*::*q35::yfp*]I), Q37 AM470 (rmIs225[P*_unc-54_*::*q37::yfp*]II) [16], [17]; human SOD1^G93A^ AM265 (rmIs177[P*_unc-54_*::*sod1^G93A^*::*yfp*]) [5]; intestinal Q44 GF80 dgEx80[pAMS66 P*vha-6*::q44::yfp + *rol-6(su1006)* +pBluescript II] [68]; temperature sensitive (TS) mutant strains CB1402 [*unc-15(e1402)*], CB1157 [*unc-54(e1157)*], HE250 [*unc-52(e669su250)*] and CB286 [*unc-45(e286)*] [4]; WM27 [*rde-1(ne219)*] and WM118 [*rde-1(ne219);neIs9[myo-3::HA::RDE-1+pRF4(rol-6(su1006))]*] [41], [42]; CB1072 [*unc-29(e1072)*], CB904 [*unc-38(e264)*], ZZ26 [*unc-63(x26)*], RB918 [*acr-16(ok789)*], VC311 [*unc-47(gk192)*], CB845 [*unc-30(e191)*], CB407 [*unc-49(e407)*] [29], MT1212 [*egl-19(n582)*], DA952 [*egl-19(n582ad952)*], DA695 [*egl-19(ad695)*] [47], [56], HK30 [*unc-68(kh30)*], PS3551 [*hsf-1(sy441)*]. Where indicated, genetic crosses between mutant animals and Q35 animals were generated.

### RNAi Assays

RNAi gene knockdown in *C. elegans* was performed as described previously, using the commercial RNAi library (GeneService, USA) [30], [69]. Briefly, animals were added to RNAi bacteria (in liquid or RNAi-seeded NGM plates) at the L1 stage (first larval, day 1), incubated at 20°C for 5 days and scored for number of aggregates at 6 days old (which corresponds to 3 days after the onset of Q35 aggregation) [30], using the stereomicroscope Leica MZ16FA (Leica Microsystems, Switzerland). Q35 aggregation was scored in at least 50 animals, for each condition (*n≥3*). As a negative control, animals were fed bacteria carrying the L4440 empty vector (vector). Liquid RNAi treatment was performed in 96-well plates, with a total volume of 60 µl per well, consisting of 15–20 worms and RNAi bacteria. Bacteria was grown overnight (∼16 h), induced with isopropyl β-D-thiogalatoside (IPTG Sigma, 1 mM for 3 h at 37°C), pelleted and resuspended in S-medium complete (S-Basal supplemented with 3 mM MgSO_4_, 3 mM CaCl_2_, 10 mM Potassium Citrate, 100 mg/ml Ampicillin and 1 mM IPTG) so that the final OD_595 nm_ was 0.9 in the well. RNAi assays on plates were performed as described previously [30], and for double knockdown experiments, equal volumes of each RNAi bacteria were mixed (1∶1 ratio) prior to plate seeding. Fluorescent microscopy images were taken using an Axiovert 200 microscope with a Hamamatsu digital camera C4742-98 (Carl Zeiss, Germany). All RNAi plasmids were sequenced to confirm correct and specific gene-target identity. Gene knockdown by RNAi was confirmed by PCR analysis.

### FRAP Analysis

Animals were mounted on a 3% (w/v) agar pad on a glass slide, immobilized with 2 mM levamisole (Sigma), and subjected to FRAP analysis using the Zeiss LSM510 confocal microscope (Carl Zeiss, Germany) as previously described [30], [70].

### Motility Assays

The movement of 6 day old animals grown on RNAi-seeded NGM plates (>75 animals per experiment, n≥3) were digitally recorded using a Leica M205 FA microscope with a Hamamatsu digital camera C10600-10B (Orca-R2, Leica Microsystems, Switzerland), and the Hamamatsu Simple PCI Imaging software. Animals were tracked using a custom ImageJ plugin wrMTrck [30]. The average speed of each animal was calculated by dividing the distance of each track, corrected for body length, by the duration (in seconds) of the track (body length per second BLPS) (*n≥3*). Results are shown relative to wt animals' speed on L4440-RNAi vector control plates (100%).

### Real-Time qPCR Analysis

RNA from ∼50 animals was extracted with Trizol (Invitrogen), followed by DNase treatment (Applied Biosystems #AM1906). mRNA was reverse transcribed using the iScript cDNA Synthesis Kit (Bio-Rad #170-8891). cDNA real-time PCR amplification was done using the iQ-SYBR Green Supermix (Bio-Rad #170-8880) and the iCycler system (Bio-Rad) (see Protocol S1). Expression levels of each gene were determined using the Comparative C_T_ Method (Real-Time PCR Applications Guide, Bio-Rad), normalized to actin (*act-1*) in the same sample, and relative to the non-treated or vector control sample. Measurements were performed for ≥3 biological samples.

### Paralysis Assays

Five day old animals grown on RNAi-seeded NGM plates at 20°C (≤40) were transferred onto NGM plates, equilibrated at 20°C, containing 1 mM Levamisole (Sigma), 30 mM Nicotine (Sigma) or the solvent (water or ethanol, respectively). Sensitivity to the drugs was followed by visual inspection every 2 to 5 min and defined as paralysis, or lack of movement in response to prodding on the nose and tail of the animal (*n = 3*). Compound stock solutions: 800 mM levamisole (Sigma) in water, 300 mM nicotine (Sigma) in ethanol.

### Compound Assays

Compound assays were performed in liquid culture as described previously [71], with 60 µl of final volume per well, 15–20 animals (in S-Basal complete), compound at the appropriate concentration and bacteria (OP50 or RNAi) at a final OD_595 nm_ of 0.9 (resuspended in S-Basal complete). Replicates of each condition were included in each assay/plate. Animals were incubated with each drug, at the concentrations indicated in the respective Figures, from L1 stage (levamisole, GABA), L2 stage (Lindane, Ryanodine, Nemadipine A, Dantrolene Sodium, 4-CmC) or L4 stage (dTBC, BAPTA), until day 6 of age, at 20°C (*n≥3*) (see Protocol S1). At this time animals were transferred from liquid culture onto NGM plates for aggregate quantification (Leica MZ16FA) and collected for real-time qPCR analysis.

### Assay for TS Phenotypes

Temperature sensitive (TS) mutant animals were age-synchronized to L1 stage, grown on RNAi-seeded NGM plates (∼20 animals per plate) from day 1 at a sensitized temperature of 23°C (to maintain the RNAi suppressor effect on aggregation), or at the control restrictive (25°C) and permissive (15°C) temperatures, and scored for phenotypes on day 5, as previously described [30]. >50 animals were scored for each TS phenotype, per assay: slow movement/paralysis assay for *unc-15(e1402)* and *unc-54(e1157)*, stiff paralysis for *unc-52(e669su250)*, and egg-laying phenotype for *unc-45(e286)* (partially paralyzed animals with a large belly of accumulated eggs) (*n = 3*) [4], [29], [30].

### Gel Mobility Shift Analysis

Native nuclear protein extracts were prepared from 200 µl of pelleted worms (grown on NGM RNAi-seeded plates), with the commercial Thermo Scientific NE-PER Nuclear and Cytoplasmic Extraction Kit (# 78835), as described previously [72]. Electrophoretic mobility shift analysis (EMSA) was performed as before [43] using a [^32^P]-labeled probe containing the proximal heat shock element (HSE) from the *C. elegans hsp-70* (*C12C8.1*) gene promoter. Nuclear extracts (40 µg) were incubated with the [^32^P]-labeled probe (HSE or mutant) for 20 min at room temperature. For heat shock treatment (HS) the samples were pre-incubated at 35°C for 30 min. For competition experiments, a 100-fold molar excess of the same unlabeled oligonucleotide was added to the mixture. The samples were analyzed by electrophoresis on a 4% (w/v) polyacrylamide native gel that was dried and scanned using a PhosphorImager (Molecular Dynamics, Sunnyvale, CA). Oligonucleotide probes: HSE-F: taaattgtagaaggttctagaagatgccaga; HSE-R: tctggcatcttctagaaccttctacaattta; HSE^mut^-F: taaattgtaaaaggaaataaaagatgccaga; HSE^mut^-R: tctggcatcttttatttccttttacaattta.

## Supporting Information

Figure S1 Suppression of protein aggregation in BWM cells by *gei-11* RNAi. (A) *gei-11* RNAi suppression of Q37 (I-IV) and SOD1^G93A^ (V-VIII) aggregation in BWM cells of 5 day old animals, shown by the diffuse fluorescent pattern in III, IV, VII and VII; in contrast to a foci-like pattern in the vector control I, II, V and VI. Boxed areas refer to magnified images to the right (scale bar 0.1 mm). (B) RT-PCR amplification of *q35-yfp* (top panel). mRNA from wt animals is a negative control for transgene amplification (left); *yfp*-RNAi is a positive control for reduced q35 mRNA levels. Actin (bottom panel) is the control for total mRNA. Band intensities were used to determine mRNA levels relative to vector control (n = 3). (C,D) SDS-PAGE and western blotting analysis of Q35 protein levels, with anti-YFP (top) and anti-α-tubulin (bottom) antibodies. Q35 protein levels relative to tubulin were calculated from band intensities on (*B*), and are shown as a relative percentage of the vector control (*n* = 3, Student t-test ***p<0.001, ±SD). (E) *gei-11* knockdown did not affect polyQ aggregation in the intestine (iQ44).
(TIF)

Figure S2 Suppression of aggregation through AChRs. (A) Suppression of Q35 aggregation by *gei-11* RNAi is abolished in the L-AChR *unc-38(e264)* mutant background. (B,C) Cholinergic sensitivity assays with 5 day old animals treated with *gei-11* or vector RNAi and scored for paralysis on (B) 1 mM Levamisole plates (±SD, two-way ANOVA ***p<0.001) and (C) on 30 mM nicotine plates (±SD; two-way ANOVA *p*>0.05 relative to vector control). N-AChR mutant *acr-16(ok789)* and L-AChR mutant *unc-38(e264)* were used as controls for receptor specificity. (D) Dose-dependent effect of AChR antagonist dTBC (in water) on Q35 aggregation (±SD). Student t-test **p<0.01 and ***p<0.001; data and statistics are relative to Q35;vector (±SD). (E) Real-time qPCR analysis of *gei-11* levels in wt, *rde-1(ne219)* and *rde-1(ne219);m*RDE-1 animals, treated with *gei-11* or vector RNAi. Data are relative to wt animals (±SD).
(TIF)

Figure S3 Effect of *gei-11* knockdown on stress responses. (A) Real-time qPCR analysis of *hsp* expression levels in heat-shock treated wt animals (45 min at 35°C, 1 h recovery at 20°C). Data are relative to wt animals in control temperature (20°C) (±SD). (B) Real-time qPCR analysis of wt animals treated with *gei-11* RNAi did not show induction of UPR-regulated ER chaperones, metabolic stress FOXO/DAF-16 regulated genes or oxidative stress regulated genes (±SD, data normalized to the levels of each gene in vector control).
(TIF)

Figure S4 Dose-dependent effect of levamisole on muscle cells Q35 aggregation. Relative foci count upon levamisole (in water) treatment, and statistics relative to Q35;vector (±SD). Student t-test **p<0.01, ***p<0.001.
(TIF)

Figure S5 Suppression of Q35 aggregation is Ca^2+^-dependent. (A,B) Suppression of Q35 aggregation and *hsp* induction by *gei-11* RNAi were prevented upon co-treatment with the Ca^2+^ chelator BAPTA (15 µM in DMSO, at L4). Data are relative to Q35;DMSO control (±SD). (C) RNAi of Ca^2+^-dependent kinases and calmodulins tested for effect on Q35 aggregation, by double RNAi with *gei-11* or vector control (±SD). Student t-test ***p<0.001.
(TIF)

Figure S6 EGL-19 and RYR agonists and antagonist dose-dependent effect on muscle cells Q35 aggregation. (A) Dose-dependent effect of Nemadipine A on suppression of Q35 aggregation by *gei-11* RNAi. Data are relative to Q35;DMSO in vector RNAi (±SD). (B,C) Dose-dependent effect of the RYR agonist 4-CmC and the antagonist DS on Q35 aggregation. % of foci are relative to Q35;water control for 4-CmC and Q35;DMSO for DS (±SD). Student t-test ***p<0.001 and **p<0.01.
(TIF)

Protocol S1 Supplementary Materials and Methods.
(DOC)

Table S1 Control values for RNAi gene knockdown.
(DOC)
