# Supplementary figures and images for: Neuronal Reprograming of Protein Homeostasis by Calcium-Dependent Regulation of the Heat Shock Response
Source: PLoS Genet. 2013 Aug 29;9(8):e1003711. doi: 10.1371/journal.pgen.1003711 (PMC3757039; doi:10.1371/journal.pgen.1003711)

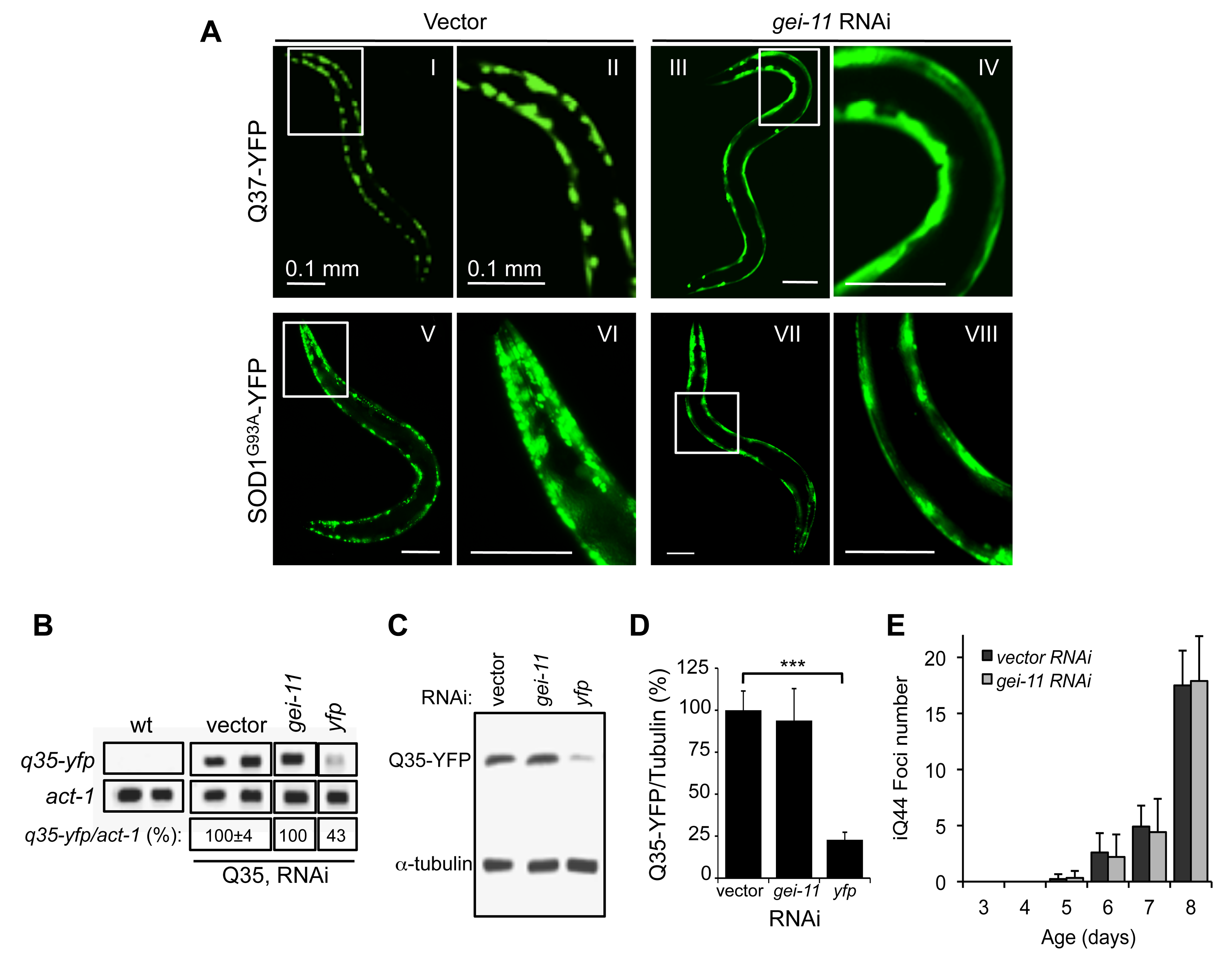

Supplement: Figure S1 — Suppression of protein aggregation in BWM cells by gei-11 RNAi. (A) gei-11 RNAi suppression of Q37 (I-IV) and SOD1G93A (V-VIII) aggregation in BWM cells of 5 day old animals, shown by the diffuse fluorescent pattern in III, IV, VII and VII; in contrast to a foci-like pattern in the vector control I, II, V and VI. Boxed areas refer to magnified images to the right (scale bar 0.1 mm). (B) RT-PCR amplification of q35-yfp (top panel). mRNA from wt animals is a negative control for transgene amplification (left); yfp-RNAi is a positive control for reduced q35 mRNA levels. Actin (bottom panel) is the control for total mRNA. Band intensities were used to determine mRNA levels relative to vector control (n = 3). (C,D) SDS-PAGE and western blotting analysis of Q35 protein levels, with anti-YFP (top) and anti-α-tubulin (bottom) antibodies. Q35 protein levels relative to tubulin were calculated from band intensities on (B), and are shown as a relative percentage of the vector control (n = 3, Student t-test ***p<0.001, ±SD). (E) gei-11 knockdown did not affect polyQ aggregation in the intestine (iQ44). (TIF) [file pgen.1003711.s001.tif]

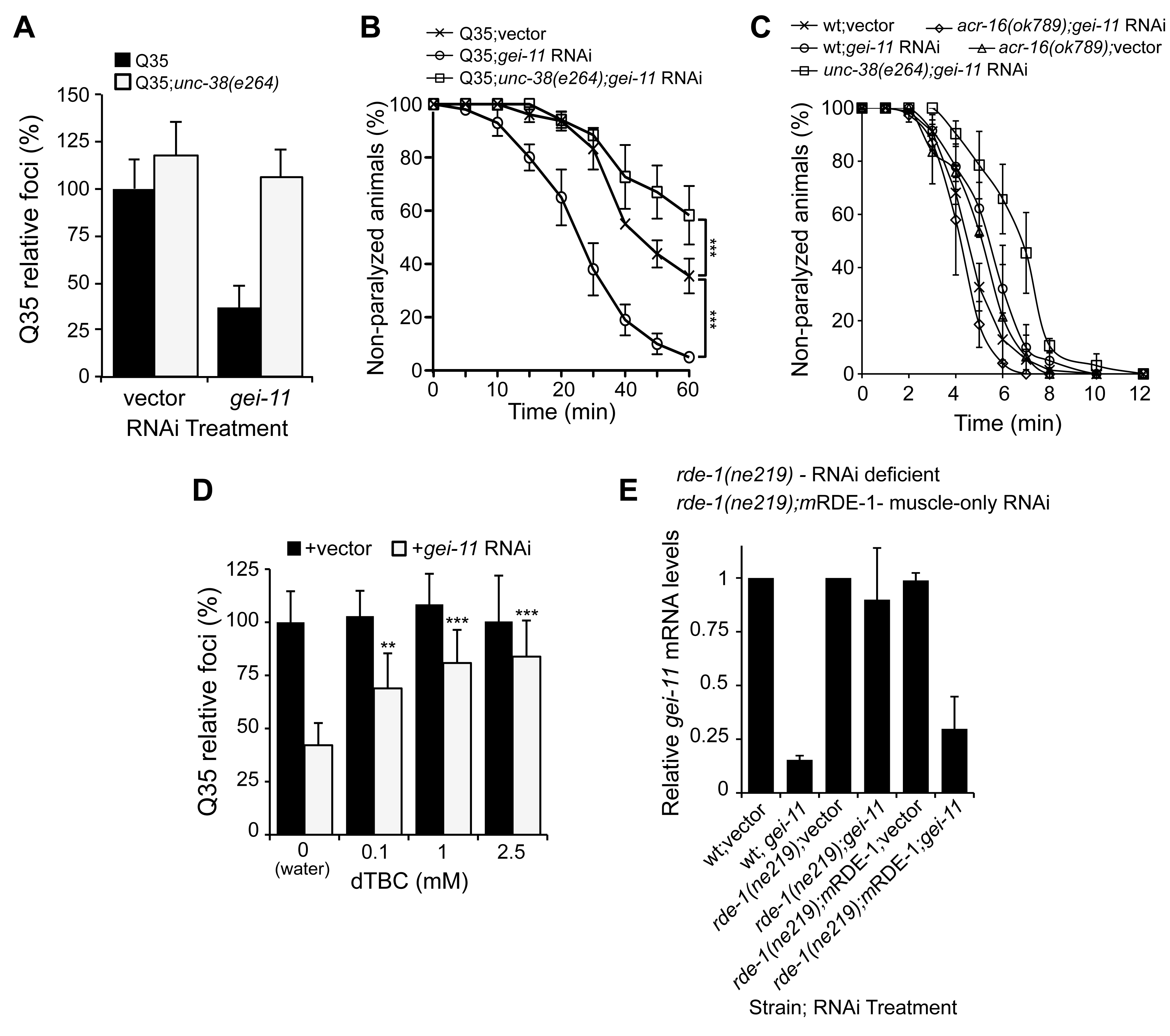

Supplement: Figure S2 — Suppression of aggregation through AChRs. (A) Suppression of Q35 aggregation by gei-11 RNAi is abolished in the L-AChR unc-38(e264) mutant background. (B,C) Cholinergic sensitivity assays with 5 day old animals treated with gei-11 or vector RNAi and scored for paralysis on (B) 1 mM Levamisole plates (±SD, two-way ANOVA ***p<0.001) and (C) on 30 mM nicotine plates (±SD; two-way ANOVA p>0.05 relative to vector control). N-AChR mutant acr-16(ok789) and L-AChR mutant unc-38(e264) were used as controls for receptor specificity. (D) Dose-dependent effect of AChR antagonist dTBC (in water) on Q35 aggregation (±SD). Student t-test **p<0.01 and ***p<0.001; data and statistics are relative to Q35;vector (±SD). (E) Real-time qPCR analysis of gei-11 levels in wt, rde-1(ne219) and rde-1(ne219);mRDE-1 animals, treated with gei-11 or vector RNAi. Data are relative to wt animals (±SD). (TIF) [file pgen.1003711.s002.tif]

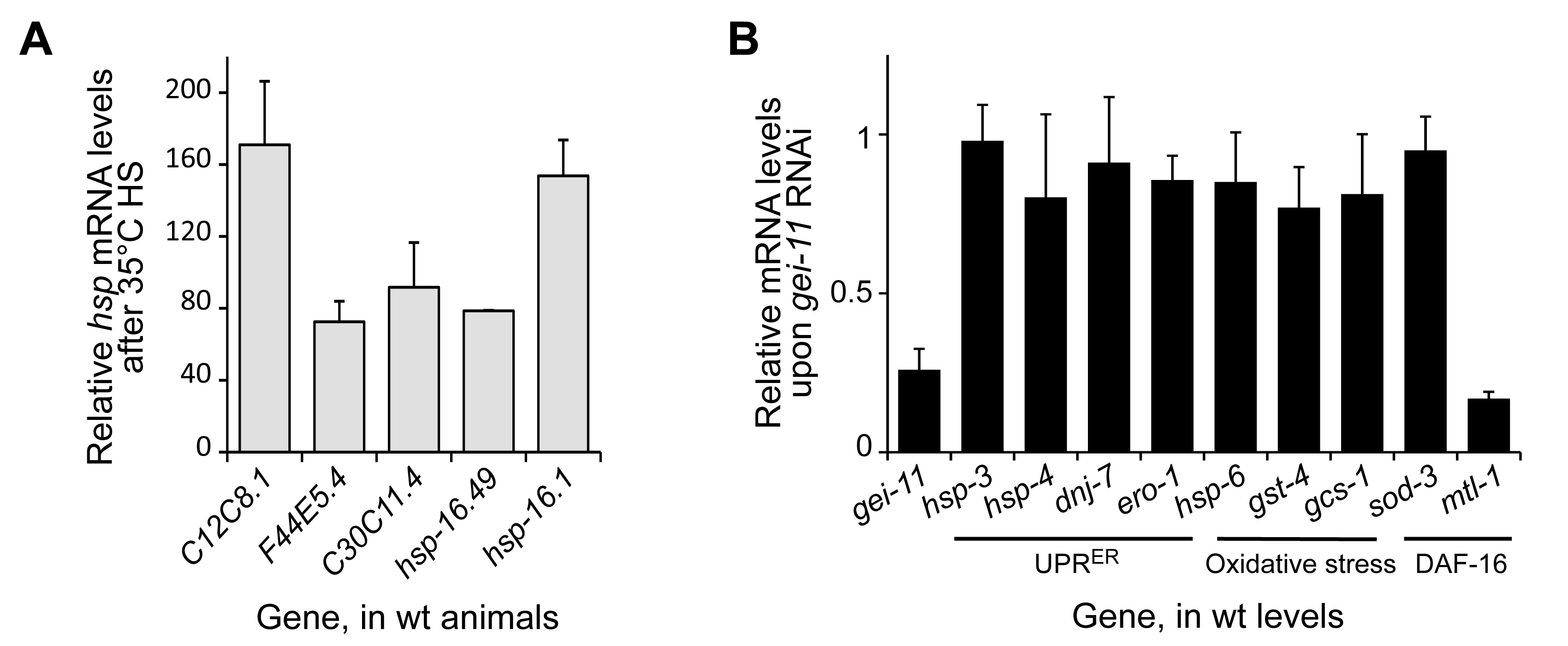

Supplement: Figure S3 — Effect of gei-11 knockdown on stress responses. (A) Real-time qPCR analysis of hsp expression levels in heat-shock treated wt animals (45 min at 35°C, 1 h recovery at 20°C). Data are relative to wt animals in control temperature (20°C) (±SD). (B) Real-time qPCR analysis of wt animals treated with gei-11 RNAi did not show induction of UPR-regulated ER chaperones, metabolic stress FOXO/DAF-16 regulated genes or oxidative stress regulated genes (±SD, data normalized to the levels of each gene in vector control). (TIF) [file pgen.1003711.s003.tif]

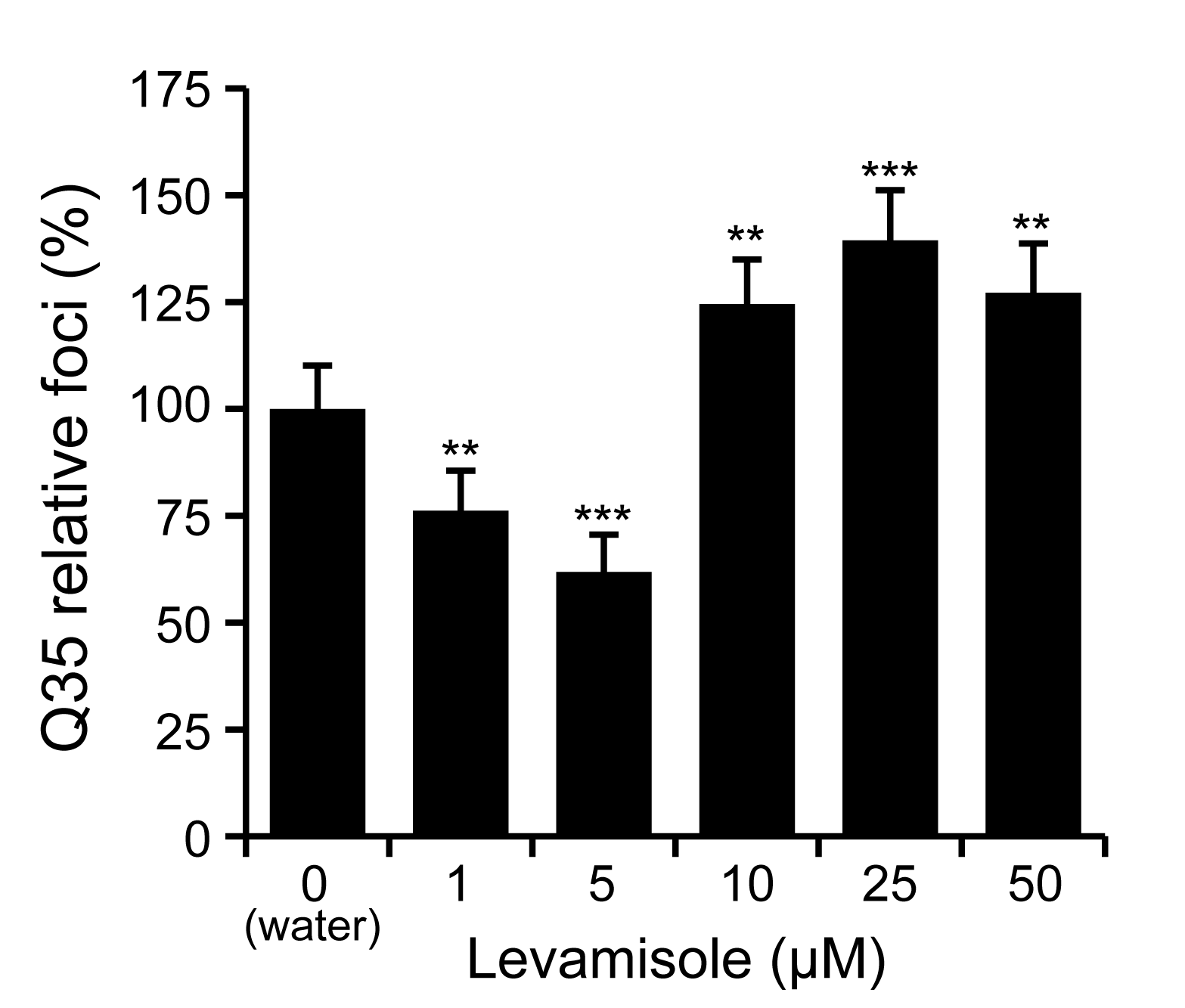

Supplement: Figure S4 — Dose-dependent effect of levamisole on muscle cells Q35 aggregation. Relative foci count upon levamisole (in water) treatment, and statistics relative to Q35;vector (±SD). Student t-test **p<0.01, ***p<0.001. (TIF) [file pgen.1003711.s004.tif]

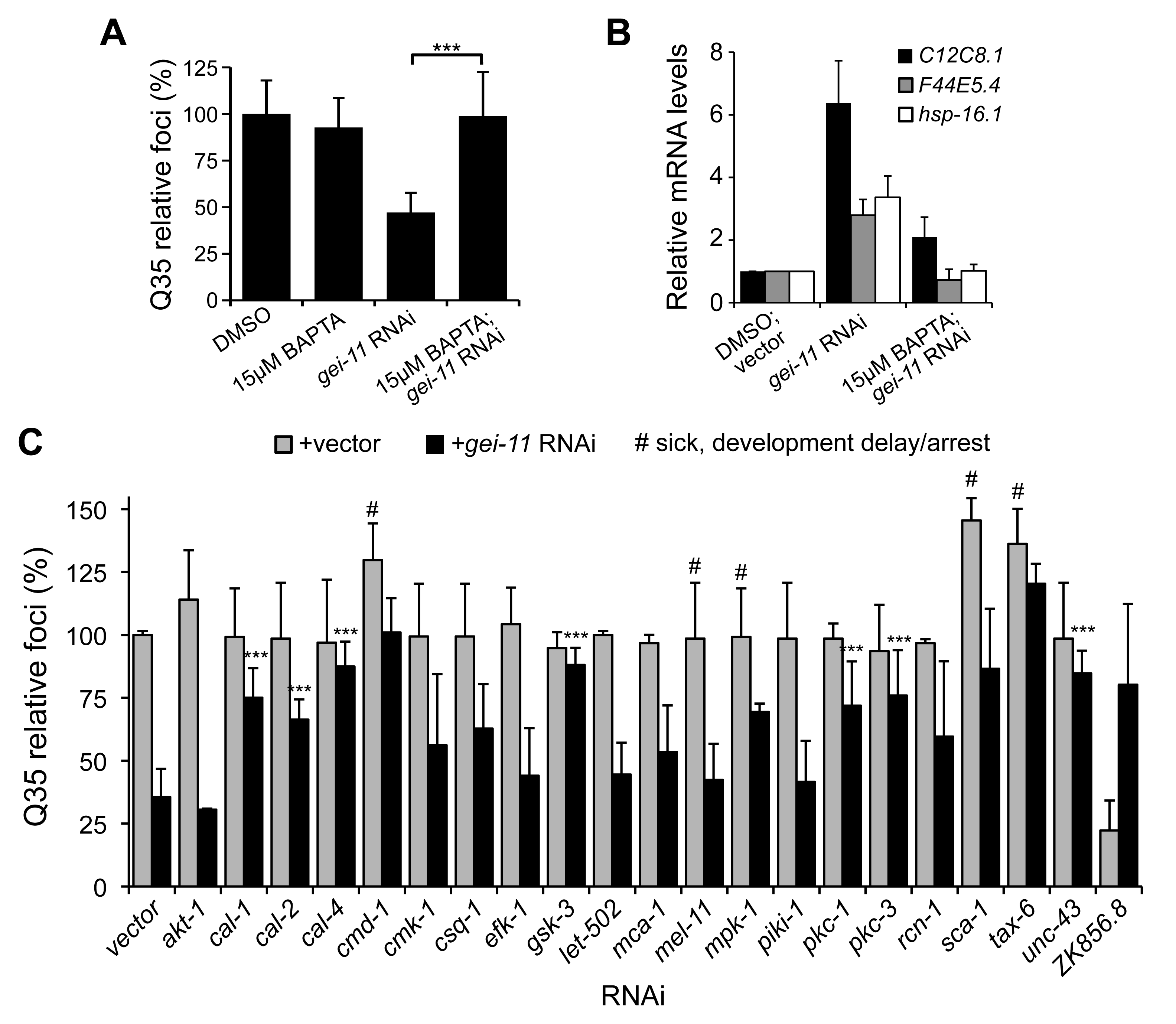

Supplement: Figure S5 — Suppression of Q35 aggregation is Ca2+-dependent. (A,B) Suppression of Q35 aggregation and hsp induction by gei-11 RNAi were prevented upon co-treatment with the Ca2+ chelator BAPTA (15 µM in DMSO, at L4). Data are relative to Q35;DMSO control (±SD). (C) RNAi of Ca2+-dependent kinases and calmodulins tested for effect on Q35 aggregation, by double RNAi with gei-11 or vector control (±SD). Student t-test ***p<0.001. (TIF) [file pgen.1003711.s005.tif]

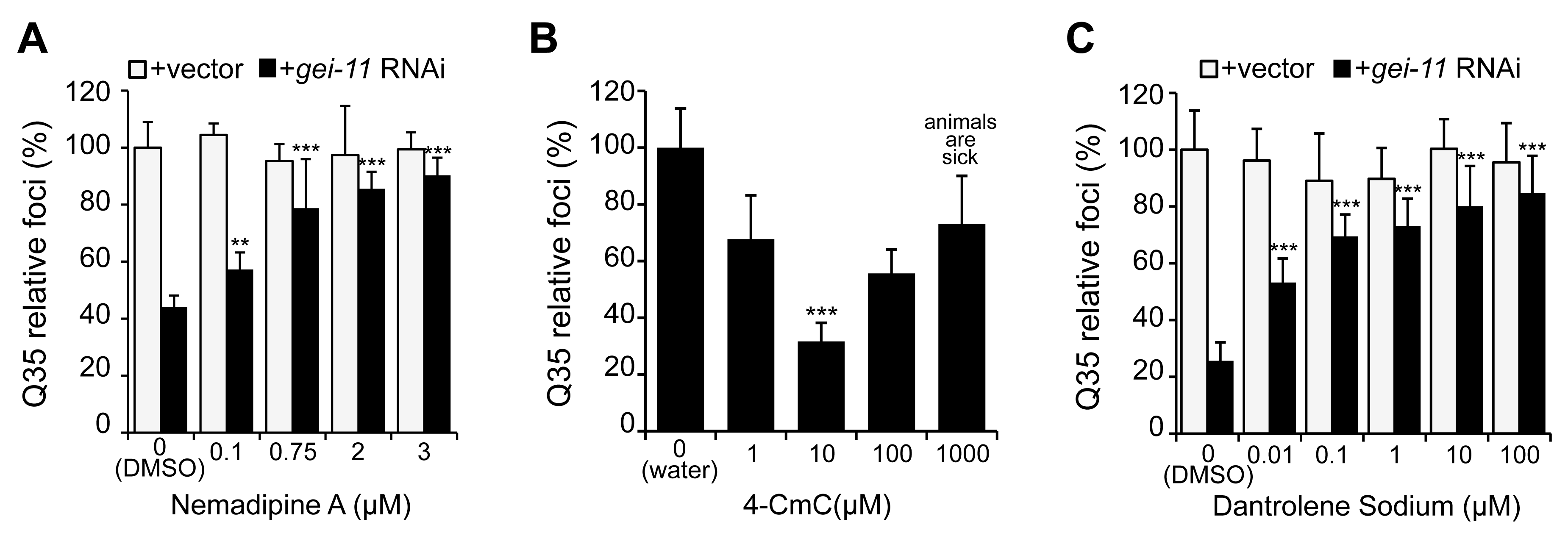

Supplement: Figure S6 — EGL-19 and RYR agonists and antagonist dose-dependent effect on muscle cells Q35 aggregation. (A) Dose-dependent effect of Nemadipine A on suppression of Q35 aggregation by gei-11 RNAi. Data are relative to Q35;DMSO in vector RNAi (±SD). (B,C) Dose-dependent effect of the RYR agonist 4-CmC and the antagonist DS on Q35 aggregation. % of foci are relative to Q35;water control for 4-CmC and Q35;DMSO for DS (±SD). Student t-test ***p<0.001 and **p<0.01. (TIF) [file pgen.1003711.s006.tif]
