# Supplementary material for: Neuronal Reprograming of Protein Homeostasis by Calcium-Dependent Regulation of the Heat Shock Response
Source: PLoS Genet. 2013 Aug 29;9(8):e1003711. doi: 10.1371/journal.pgen.1003711 (PMC3757039; doi:10.1371/journal.pgen.1003711)
Supplement: Protocol S1 — Supplementary Materials and Methods. (DOC) [file pgen.1003711.s007.doc]

**Protocol S1:** Supporting Materials and Methods

**PCR Analysis**

Animal lysis and RNA extraction were accomplished by vortexing with Trizol (Invitrogen). Chloroform was added to each sample, followed by vortexing and centrifugation at 13,500x*g* for 15 min (4°C). 2-Propanol was added to the aqueous layer and total RNA was spun down at 18,000xg (4°C) for 10 min. RNA pellets were washed with 75% (v/v) ethanol, air-dried and resuspended in nuclease free water. RNA aliquots of 10 µg were used for DNase treatment (Applied Biosystems #AM1906), and purified RNA was used for cDNA synthesis (Bio-Rad #170-8891). For real time PCR analysis, 10 ng of cDNA were used for PCR amplification with specific primers for each gene (see list below) and the iQ SYBR Green Supermix (Bio-Rad #170-8880), in the iCycler system (Bio-Rad).

For standard PCR, cDNA samples were amplified with the following primers:

actin.F 5’-ATCACCGCTCTTGCCCCATC-3’;

actin.R 5’-GGCCGGACTCGTCGTATTCTTG-3’;

Q35-yfp.F 5’-GCTGCCCGACAACCACTAC-3’;

Q35-yfp.R 5’-TGGCGATCTGATGACAGCGG-3’.

Amplified products were ran on a 1% (w/v) agarose-TBE gel with ethidium bromide, and scanned with the UV scanner Alpha Imager EC (Alpha Innotech).

Primers used for real-time qPCR:

*gei-11*.F ctctcgatgcatcggttaagc; *gei-11*.R gttgttcgtcttcaaccagc;

*unc-29*.F gaggaccaaccgactatcatgg; *unc-29*.R cacattgatgagcaacaccaac;

*unc-38*.F catgggatccagtgaattatggg; *unc-38*.R atactcttgaagattgctggtg;

*unc-63*.F ggaccaaatgaccacggttttgc; *unc-63*.R gaacgtcaccacgagtgtctctc;

*acr-16*.F ctcatctcgtgcgcaattcttgc; *acr-16*.R cgtcaattatttgttggagggc;

*unc-49*.F gttgccgcagtttgtacttcagtc; *unc-49*.R cgacaatcaggacagatggtag;

*lev*-8.F ggagagccacatatcacaatcac; *lev*-8.R gtttctccgtcattcgaccatcc;

*C12C8.1*.F actcatgtgcggtatttatca; *C12C8.1*.R acgggctttccttgtttt;

*C30C11.4*.F gcttcgtactattgtggaatctc; *C30C11.4*.R ggagatcttgcttgtagattcc;

*F44E5.4*.F gtcttgatgcaaagctattggtatc; *F44E5.4*.R cgtcgtccaatcaatccttttgcatc;

*hsp-16.1.*F actttaccactatttccgtccagc; *hsp-16.1.*R gataatgtatgtccatccaaatta;

*hsp-12.6*.F atgatgagcgttccagtgatggctgacg; *hsp-12.6*.R ccatgtgaatccaagttgctcttg;

*hsp-16.49*.F ccatattggagaaatgctgatcac; *hsp-16.49*.R cttctggtagccgcctcattttg;

*mtl-1*.F ggcttgcaagtgtgactgcaaaaacaagc; *mtl-1*.R ttaatgagccgcagcagttccctggtgttgatgg; *sod-3*.F gcttcaaagcttgttcaaccggttgcg; *sod-3*.R cagcgctggttggagagcaattgc;

*hsp-6*.F caggccgttaccaactctgc; *hsp-6*.R gcagtttccttcatcttcatc;

*gcs-1*.F gtgcaagtgtcgacgatcgtac; *gcs-1*.R gcgaatatgttttgccagtggctc;

*gst-4*.F cgactccatttggccagc; *gst-4*.R gatcagcgtcacttctag;

*hsp-4*.F gcagatgatcaagcccaaaaag; *hsp-4*.R ggagacgattggttgaacaacag;

*hsp-3*.F cctacggacttgacaagaaggac; *hsp-3.*R gaacggcacgattgtctttgc;

*dnj-7*.F gagcacggatagtacggac; *dnj-7*.R gaggcaagccatccagtc;

*ero-1*.F cgacgaaatgcagtgaatatg; *ero-1*.R gtgtagcgttccggattcttcg;

**Compound Assays**

Assays were performed in 96-well plates with 15-20 animals per well in a total volume of 60 μl, compound at the appropriate concentration and bacteria (OP50, vector RNAi or *gei-11* RNAi bacteria). RNAi bacteria grown overnight (~16h) was induced with IPTG (1 mM for 3.5 h at 37°C), pelleted and resuspended in S-medium complete (S-Basal supplemented with 3 mM MgSO_4_, 3 mM CaCl_2_, 10 mM potassium citrate, 100 mg/ml Ampicillin and 1 mM IPTG) so that the final OD_595nm_ is 0.9 in the well. Compound stock solutions: 500 mM GABA (Sigma) in water; 1 mM Levamisole (Sigma) in water; 200 mM (+)-Tubocurarine Chloride (D-Tubocurare/dTBC, Sigma) in water; 100 mM Lindane (Sigma) in 10% ethanol; 5 mM Ryanodine (Tocris Bioscience) in 100% ethanol; 1 mM Nemadipine A (Nem.A, Sigma) in 100% DMSO (dilutions in water); 5mM BAPTA (Invitrogen) in 100% DMSO; 10mM Dantrolene Sodium (DS, Sigma) in 100% DMSO; 20mM 4-Chloro-m-cresol (4-CmC, Supelco Analytical) in water. Concentrations used per experiment can be found in the respective Figures and legends.

**SDS-PAGE and Western Blotting Analysis**

Six day old animals (50 to 100 animals) grown on RNAi-seeded NGM plates at 20°C, were collected with M9 buffer and resuspended in PELE buffer for lysis, protein extraction and analysis by SDS-PAGE and western blotting were as previously described . Antibody binding was detected with the Odyssey Infrared Imaging System (LI-COR Biosciences, USA) and intensity of the bands were measured using Adobe Photoshop 7.0. To determine relative Q35 protein levels, the ratio YFP/α-tubulin between band intensities was calculated (arbitrary units) and compared to the EV control sample (*n≥3).*

**References**

1. Silva MC, Fox S, Beam M, Thakkar H, Amaral MD, et al. (2011) A Genetic Screening Strategy Identifies Novel Regulators of the Proteostasis Network. PLoS Genet 7: e1002438.
