# Supplementary material for: Neuronal Reprograming of Protein Homeostasis by Calcium-Dependent Regulation of the Heat Shock Response
Source: PLoS Genet. 2013 Aug 29;9(8):e1003711. doi: 10.1371/journal.pgen.1003711 (PMC3757039; doi:10.1371/journal.pgen.1003711)
Supplement: Table S1 — Control values for RNAi gene knockdown. (DOC) [file pgen.1003711.s008.doc]

**Table S1:** **Control values for RNAi gene knockdown.**

Gene levels (average ± SD; *n*≥3) upon gene-RNAi. Values shown are relavant for the Figures indicated in the first column. (1:1) refers to gene-RNAi dilution with vector or *gei-11* RNAi.

|  | **Gene/RNAi** | **Gene levels relative to vector control** |
| --- | --- | --- |
| (Fig.2B) | *gei-11* | 0.18±0.017 (1:1) |
|  | *unc-38* | 0.27±0.014 (1:1) |
|  | *unc-63* | 0.54±0.207 (1:1) |
|  | *unc-29* | 0.43±0.102 (1:1) |
|  | *lev-1* | 0.31±0.082 (1:1) |
|  | *acr-16* | 0.42±0.293 (1:1) |
| (Fig.3B) | *gei-11* | 0.17±0.030 (1:1) |
|  | *hsf-1* | 0.38±0.140 (1:1) |
| (Fig.4C) | *unc-49* | 0.31±0.014; 0.43±0.202 (1:1) |
|  | *unc-47* | 0.56±0.403; 0.67±0.200 (1:1) |
| (Fig.6B) | *egl-19* | 0.36±0.071 (1:1) |
|  |  |  |
